# Aristolochic acid I induces proximal tubule injury through ROS/HMGB1/mt DNA mediated activation of TLRs

**DOI:** 10.1111/jcmm.17451

**Published:** 2022-06-28

**Authors:** Rohit Upadhyay, Vecihi Batuman

**Affiliations:** ^1^ Section of Nephrology and Hypertension, John W. Deming Department of Medicine Tulane University School of Medicine New Orleans Louisiana USA; ^2^ Medicine Service, Section of Nephrology Southeast Louisiana Veterans Health Care System (SLVHCS) New Orleans Louisiana USA

**Keywords:** aristolochic acid I, kidney injury, mitochondrial dysfunction, ROS, TLRs

## Abstract

Aristolochic acids (AAs) are extracted from certain plants as folk remedies for centuries until their nephrotoxicity and carcinogenicity were recognized. Aristolochic acid I (AAI) is one of the main pathogenic compounds, and it has nephrotoxic, carcinogenic and mutagenic effects. Previous studies have shown that AAI acts mainly on proximal renal tubular epithelial cells; however, the mechanisms of AAI‐induced proximal tubule cell damage are still not fully characterized. We exposed human kidney proximal tubule cells (PTCs; HK2 cell line) to AAI in vitro at different time/dose conditions and assessed cell proliferation, reactive oxygen species (ROS) generation, nitric oxide (NO) production, m‐RNA/ protein expressions and mitochondrial dysfunction. AAI exposure decreased proliferation and increased apoptosis, ROS generation / NO production in PTCs significantly at 24 h. Gene/ protein expression studies demonstrated activation of innate immunity (TLRs 2, 3, 4 and 9, HMGB1), inflammatory (IL6, TNFA, IL1B, IL18, TGFB and NLRP3) and kidney injury (LCN2) markers. AAI also induced epithelial‐mesenchymal transition (EMT) and mitochondrial dysfunction in HK2 cells. TLR9 knock‐down and ROS inhibition were able to ameliorate the toxic effect of AAI. In conclusion, AAI treatment caused injury to PTCs through ROS‐HMGB1/mitochondrial DNA (mt DNA)‐mediated activation of TLRs and inflammatory response.

## 1 INTRODUCTION

Aristolochic acids (AAs) are naturally occurring polyaromatic nitrogen compounds extracted from certain plants known as Aristolochia (birthworts or pipevines) and some types of plants known as Asarum (wild ginger), which grow worldwide. Plants containing aristolochic acids were used in some herbal remedies to treat a variety of symptoms and diseases, such as arthritis, gout and inflammation for centuries^1^, ^2^ until their nephrotoxicity^3^, ^4^ and carcinogenicity^5^, ^6^ began to be recognized. The ingestion of herbal products containing AAs can lead to rapid development of a chronic, progressive interstitial kidney disease, often associated with subsequent development of cancer of the upper urothelial tract.^7^ This clinical syndrome was recognized initially among women who developed severe renal disease after ingesting slimming pills containing *Aristolochia fangchi* in a weight reduction clinic in Belgium.^3^, ^8^ The kidney lesions demonstrated progressive atrophy of renal proximal tubules and extensive interstitial fibrosis involving mostly the outer renal cortex. The glomeruli were spared, and there was only minimal inflammatory cell infiltration.^7^, ^9^ On follow‐up, many patients developed upper urinary tract uroepithelial cancer.^5^, ^10^ The incidence of AA‐induced nephropathy (AAN) is not limited to any specific geographic region and cases occur regularly throughout the world, despite warnings from many health agencies. Kidney injury and fibrosis are predictable features of AAN, such that it is now used as a kidney injury/fibrosis model in vitro and in vivo studies.^11^, ^12^, ^13^ Although studies have provided considerable insights into mechanisms of AA‐induced nephropathy, the pathophysiology of AAN remains incompletely understood.^14^, ^15^, ^16^, ^17^, ^18^, ^19^

The toll‐like receptors (TLRs) are pattern recognition receptors (PRRs) that have a unique and essential function in innate immunity. There are ten known TLRs in humans^20^ which could be on cell surface/ plasma membrane (TLRs 1,2,4,5 and 6) or in the membranes of endosomes/ lysosomes (TLRs 3, 7, 8 and 9). These TLRs have specific ligands which could be alien (bacterial, viral, fungal, synthetic, etc.) or endogenous in origin (host‐originated damage‐associated molecular patterns [DAMPs] like HMGB1, histones and mtDNA). Endogenous damage signals can stimulate an inappropriate innate immune response that can initiate a pro‐inflammatory cytokine cascade.^21^, ^22^ The presence of extensive fibrosis suggests a significant role for inflammatory pathways. Specifically, the role of TLRs, mediators of innate immunity, is unknown. Since the TLRs act as first responders for danger signals and regulate downstream inflammatory and profibrotic cytokines, we investigated the role of TLRs in AAI‐induced HK‐2 cell damage.

## 2 MATERIALS AND METHOD

### 2.1 Chemical

**FIGURE 1:**
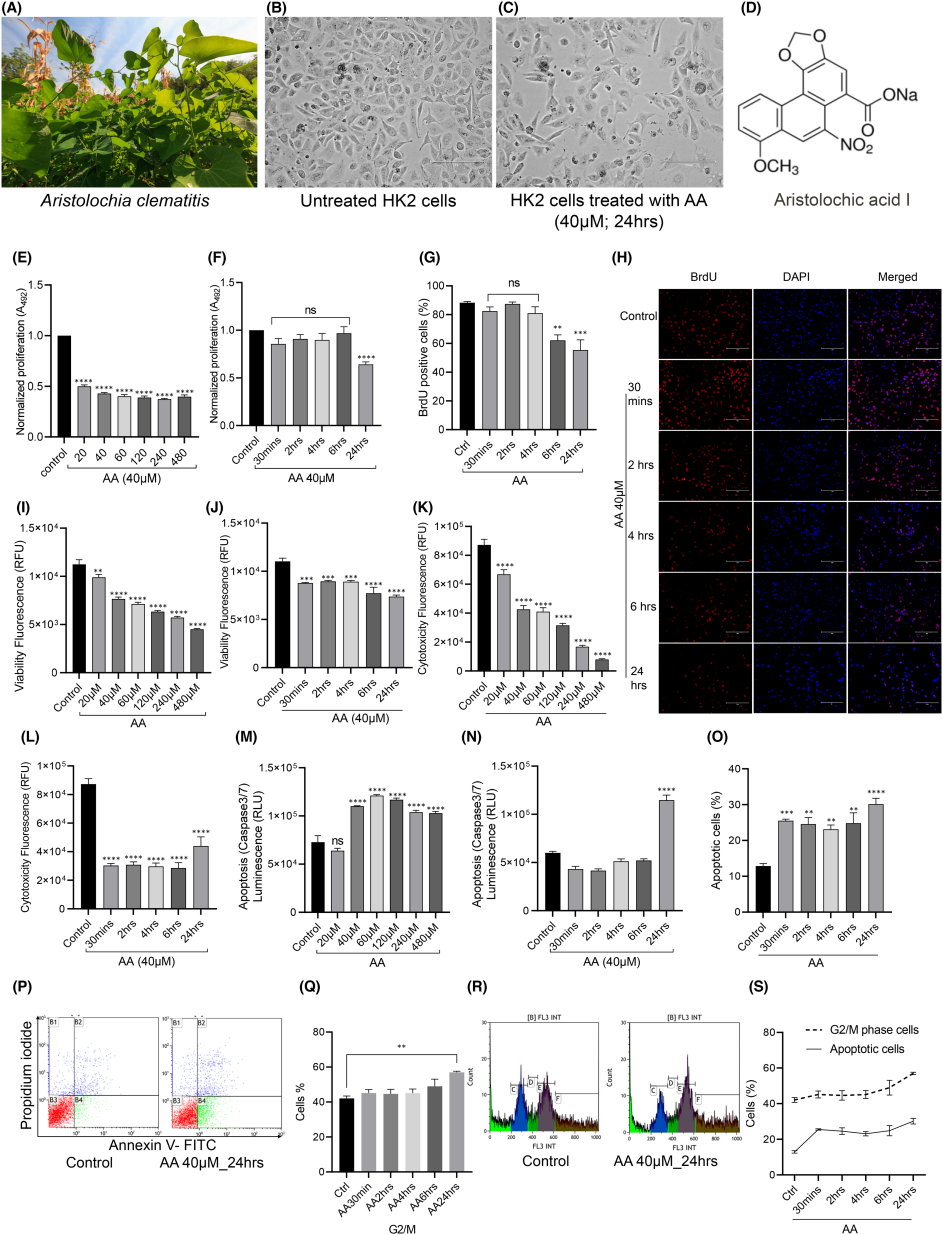
: Aristolochic Acid I (AAI) exposure caused toxicity in HK2 proximal tubule cells. A: *Aristolochia clematitis*, a plant that contains AAI; B: Morphology of HK2 cells: Untreated vs AAI (40 μM) treated for 24 h. (C); D: Chemical structure of AA; E: Dose response of AA on the proliferation of HK2 cells at 24 h.; F: Time response of AAI (40 μM) on proliferation of HK2 cells. One‐way anova ****p < 0.0001, *n* = 6; G: BrdU‐positive cell percentage in control vs AA (time response). One‐way anova **p = 0.0015, ***p = 0.0001, *n* = 4; H: Fluorescence images showing BrdU‐positive cells and DAPI stained nuclei of HK2 cells control vs AA‐exposed cells (time response); I: Viability (dose response); J: Viability (time response); K: Cytotoxicity (dose response); L: Cytotoxicity (time response); M: Apoptosis (Caspase 3/7): dose response; N: Apoptosis (Caspase 3/7): time response; O: Percentage of apoptotic cells (early and late apoptotic cells) identified by flow cytometry through propidium iodide and Annexin V‐FITC staining (P); Q: Percentage of cells in G2/M stage; One‐way anova **p = 0.0052, *n* = 3; R: Cell cycle analysis graphs in control vs AA (40 μM) exposed HK2 cells; S: superimposed graphs of percentage of cells undergoing G2/M stage arrest and apoptosis

We purchased aristolochic acid I (Sigma, catalogue A9451) (purity ≥97%, CAS: 10190–99‐5, PubChem CID: 154914, Lot number: WXBD0063V) from Sigma‐Aldrich, MO, USA). The chemical structure of AAI is shown in Figure 1D.

### 2.2 Human proximal tubule cell culture

We used human kidney proximal tubule cell line HK2 [ATCC, catalogue CRL‐2190] to evaluate the effect of AAI. We cultured and prepared cells for experiments as previously described.^23^, ^24^, ^25^, ^26^ Briefly, HK2 cells were grown (passage <10) on CellBIND surface flasks/6‐well plates (Corning) and incubated at 37°C with 5% CO2 in keratinocyte serum‐free medium (catalogue 10744019) (Gibco, Invitrogen, Thermo Fisher Scientific) supplemented with recombinant human epidermal growth factor (5 ng/mL) and bovine pituitary extract (50 μg/mL) and exchanged media at 2–3 days of intervals. We exposed cells to AAI at varying doses and time intervals. We collected cell supernatants for ELISA and cell pellets for gene expression studies. Based on our dose‐time screening data, we used a concentration of AAI (40 μM) for an exposure time of 24 h for most of the experiments.

### 2.3 Cell proliferation assay

We used CellTiter 96 AQueous One Solution Cell Proliferation Assay (MTS) (Promega, catalogue G3580) to check proliferation of HK2 cells. We seeded the cells with the density of 10 K cells per well in 96‐well plates and exposed them to AAI for the indicated time periods followed by the MTS assay according to the manufacturer's instructions.

### 2.4 Bromodeoxyuridine (BrdU) assay

AAI (40 μM) exposed HK2 cells at different time points (30 mins, 2, 4, 6 and 24 h) and untreated cells were incubated with 10 μg/mL BrdU for 40 mins at 37°C. Cells were washed with PBS and fixed in 4% PFA. Antigen retrieval was done in 2NHCL, and cells were blocked with 5% donkey serum. Anti‐BrdU primary antibody (1:200, Novus Biologicals, catalogue NBP2‐14890) at 4°C overnight and goat anti‐rabbit IgG (H + L) Superclonal Secondary Antibody, Alexa Fluor 555 (Thermo Fisher Scientific, catalogue A27039) were used to stain cells followed by mounting on glass slides with UltraCruz Aqueous Mounting Medium with DAPI (Santa Cruz Biotechnology, catalogue sc‐24,941). We captured images using fluorescent microscope (M5000, Thermo Fisher Scientific; Pittsburgh, PA, USA) and used ImageJ software to quantitate and analyse IF images through counting BrdU‐positive cells.

### 2.5 ApoToxGloTM triplex assay

We purchased the ApoToxGloTM Triplex Assay from Promega (Fitchburg, WI, USA, catalogue G6320) and performed according to the manufacturer's instructions.

### 2.6 Flow cytometry

We used Beckman coulter kit for propidium iodide (PI) staining and Annexin V FITC kit (sigma) for apoptosis assay. We followed kit protocols as per manufacturer's instructions. For analysing flow cytometry data, we used ModFitLT software (verity Software House, ME, USA) and Kaluza flow analysis software (Beckman Coulter, Inc., CA, USA). Cell cycle and apoptosis were analysed in control and AA‐exposed samples at different time intervals.

### 2.7 Real‐Time quantitative polymerase chain reaction (qPCR)

We isolated total RNA from cultured HK2 cells using the RNeasy Plus Mini Kit (QIAGEN, catalogue 74,136). Total RNA (1 μg) of purified RNA was used to prepare cDNA through high‐capacity RNA to cDNA kit (Applied Biosystems, catalogue 4,368,814). For candidate gene expressions, we used the fluorescent dye SYBR Green methodology and CFX96 Touch Real‐Time PCR machine (Bio‐Rad). We purchased the bioinformatically validated primer sets (QuantiTect Primer Assays, QIAGEN, or IDT) for use in SYBR Green‐based RT‐PCR and measured real‐time fluorescence from SYBR Green (Applied Biosystems) by the Bio‐Rad CFX Manager 3.1 System Software. We normalized gene expression to the endogenous controls (β‐actin) and estimated comparative CT values. We calculated relative gene expression through 2^–(ΔΔCT)^ method and expressed in arbitrary units (a.u.) relative to paired controls. We generated heatmaps of gene expressions by using the ‘Heatmapper’ web server.^27^

### 2.8 Western blotting

We isolated protein from cells and quantified it by using Pierce BCA Protein Assay Kit (Thermo Fisher Scientific, catalogue 23,225). We used immunoblotting to detect the expression of proteins with antibodies against TLR2 (dilution 1:200; Santa Cruz Biotechnology, catalogue sc‐21,759), TLR4 (dilution 1:200; Santa Cruz Biotechnology, catalogue sc‐293,072), TLR6 (dilution 1:1000; ProSci, catalogue 3651), TLR9 (dilution 1:1000; Cell Signalling Technology, catalogue 5845) and β‐actin (dilution 1:1000; LI‐COR Biotechnology, catalogue 926–42,212). We separated equal amounts of proteins (10–20 μg) through Bolt 4%–12% Bis‐Tris Gel (Thermo Fisher Scientific, catalogue NW04122BOX) electrophoresis and transferred onto nitrocellulose membrane (LI‐COR Biotechnology, catalogue 926–31,092). We blocked the membranes with PBS, TBS or 5% milk in Tris‐Buffered Saline‐Tween (0.05%) solution based on primary antibody‐specific recommendations of the manufacturer. After overnight incubation with primary antibody, we incubated the blots with LI‐COR secondary antibody (Odyssey IRDye 680RD, catalogue 926–68,070 or 800CW, catalogue 926–32,211) for 1 h. We visualized Western blots by Odyssey CLx Imaging System (LI‐COR Biotechnology) using near‐infrared fluorescence capture. We performed Western blot image analysis using Image Studio software (LI‐COR Biotechnology) to obtain the integrated intensities. We analysed data after normalizing with endogenous control protein (β‐actin).

### 2.9 ELISA

We determined the levels of IL6 (Human IL‐6 ELISA Ready‐SET‐Go, eBioscience, San Diego, CA, USA, catalogue 88–7066) and KIM1 (Human Urinary TIM‐1/KIM‐1/HAVCR Quantikine ELISA Kit; R&D Systems, MN, USA, catalogue DKM100) by ELISA in the cell culture medium according to the manufacturer's instructions.

### 2.10 Immunofluorescence

We performed immunofluorescence microscopy using 4% paraformaldehyde‐fixed HK2 cells. We permeabilized cells with Triton X‐100 (0.1%) and BSA (2%) in PBS for intra‐cellular proteins but not for cell surface proteins. We blocked cells in normal goat serum (Cell Signalling Technology, catalogue 5425S) plus 0.1% Triton X‐100 (PBST) for 1 h. We incubated slides in LCN2 primary antibody (dilution 1:100; Abcam, catalogue Ab63929); TLR9 (Cell signalling Technology, catalogue 13,674), NOS2 (Santa Cruz Biotechnology, catalogue sc‐7271), GRP75 (Cell Signalling Technology, catalogue 3593), MTCO2 (Invitrogen, catalogue A‐6404), CHOP (Cell Signalling Technology, catalogue 2895), Beclin 1 (Cell Signalling Technology, catalogue 3495), LC3AB (Cell Signalling Technology, catalogue 12,741), GRP78 (Cell Signalling Technology, catalogue 3177), a‐SMA (Millipore Sigma, catalogue A5228), E‐cadherin (Santa Cruz Biotechnology, sc‐8426) and cleaved caspase 3 (Cell Signalling Technology, catalogue 9661) that were diluted in blocking buffer overnight at 4°C. We washed cells in PBST three times and used goat anti‐rabbit IgG (H + L) Superclonal Secondary Antibody, Alexa Fluor 555 (Thermo Fisher Scientific, catalogue A27039) or Goat anti‐Mouse IgG (H + L) Secondary Antibody, DyLight 488 (Thermo Fisher Scientific, catalogue 35,502) at a concentration of 1.0 μg/mL (1:1000 or 1:300 for antibodies raised in rabbit or mouse, respectively) in PBS containing 0.2% BSA for 1 h at room temperature. As a non‐specific protein staining, we used Phalloidin DyLight 488 (green; catalogue 21,833) or DyLight 554 (red; catalogue 21,834) following manufacturer's instructions. We then washed the samples in PBST and rinsed in phosphate buffer; no. 1.5, mounted coverslips with UltraCruz Aqueous Mounting Medium with DAPI (Santa Cruz Biotechnology, catalogue sc‐24,941). We captured images on original magnification, 20X or 40X, using fluorescent microscope (M5000, Thermo Fisher Scientific; Pittsburgh, PA, USA). We used ImageJ software to quantitate and analyse IF images through corrected total cell fluorescence (CTCF) values.

### 2.11 Reactive oxygen species (ROS) generation assay

We estimated general oxidative stress and production of ROS in cells using the 5 μM of the redox‐sensitive fluorescent probe chloromethyl derivative of 2′,7′‐dichlorodihydrofluorescein diacetate (CM‐H2DCFDA; catalogue #c6827, Thermo Fisher Scientific; Pittsburgh, PA, USA). This probe freely enters the cell and is hydrolysed by intracellular esterase into its non‐fluorescent form 2′,7′‐dichlorofluorescin (DCFH). In the presence of ROS, DCFH is oxidized into the highly fluorescent compound DCF (2′,7′‐dichlorofluorescein), which is no longer membrane permeant. We seeded HK2 cells (3 × 10^5^) and allowed to attach for 24 h in 6‐well plates. After replacing culture media by fresh media, we exposed cells to AAI (40 μM for 2, 6 and 24 h) or H2O2 (50 μM for 10 min) or tempol (1 mM, Enzo Life Sciences, Farmingdale, NY, catalogue NC0748955). Control treatment consisted of media without AAI. After incubation for designated time periods, we washed cells with HBSS and incubated for 30 min with 5 μg/mL DCFDA and washed again with HBBS. We then added fresh PBS (100 μL) and detected DCF using the fluorescent microscope (M5000, Thermo Fisher Scientific; Pittsburgh, PA, USA). CTCF values were calculated as described previously.

### 2.12 Nitric oxide (NO) production assay

We measured NO production by loading HK2 cells with the membrane permeable NO‐sensitive dye 4‐Amino‐5‐methylamino‐2′,7′‐difluorofluorescein (DAF‐FM; Thermo Fisher Scientific; Pittsburgh, PA, USA catalogue D23841) diacetate (5 μM) for 30 min at 37 °C, 5% CO2 saturated humidity and washed in HBSS for three times. DAF‐FM is a reagent used to detect and quantify low concentrations of nitric oxide (NO). It is essentially non‐fluorescent until it reacts with NO to form a fluorescent benzotriazole. DAF‐FM fluorescence was measured by using a Bio‐tek Synergy™ HT Multi‐Detection Microplate Reader (Biotek®, Winooski, VT, USA) through excitation at 480 nm and emission at 535 nm wavelength and normalized with total protein.

### 2.13 TMRE assay

To quantify changes in mitochondrial membrane potential in HK2 cells, we used TMRE‐Mitochondrial Membrane Potential Assay Kit (Abcam catalogue ab113852) and measured fluorescence by microplate spectrophotometry. To label active mitochondria, we used TMRE (tetramethylrhodamine, ethyl ester, which is a cell permeant, positively charged, red‐orange dye that readily accumulates in active mitochondria due to their relative negative charge. Depolarized or inactive mitochondria have decreased membrane potential and fail to sequester TMRE. As a positive control, we used FCCP (carbonyl cyanide 4‐[trifluoromethoxy] phenylhydrazone), which is an ionophore uncoupler of oxidative phosphorylation. FCCP eliminates mitochondrial membrane potential and TMRE staining. We seeded HK2 cells (20 × 10^3^) and allowed to attach for 24 h in 96‐well plates. We then replaced the culture media by fresh media with or without AAI at different concentrations for 24 h. FCCP was added to appropriate control cell samples at the end of experiment, and cells were incubated for 10 min. After washing cells with PBS (1X), we incubated them with TMRE for 15–30 min, washed again twice with PBS and then analysed using Bio‐tek Synergy™ HT Multi‐Detection Microplate Reader (Biotek®, Winooski, VT, USA) at Ex/Em 549/575 nm.

### 2.14 Transfection with siRNA

To knock‐down the expression of TLR9 gene, we used TLR9 siRNA (catalogue AM16708; Assay Id 108,946, Ambion, Thermo Fisher Scientific; Pittsburgh, PA, USA). As a control for transfection, we used control siRNA‐A (catalogue sc‐37,007, Santa Cruz Biotechnology Inc.), which consists of a scrambled sequence. To transfect HK2 cells with TLR9siRNA, we used TransIT‐X2® Dynamic Delivery System (Mirus Bio LLC, Madison, WI, USA, catalogue MIR 6003).

### 2.15 Statistical analysis

We analysed data by comparing mean values through either unpaired *t*‐test (two‐tailed, for comparing 2 groups), ratio paired *t*‐test (for ELISA results) or one‐way anova (for comparing multiple groups) with Dunnett's multiple comparisons post hoc test. *p* values <0.05 were assumed significant. Data were expressed as mean ± standard error of mean (S.E.M.). We analysed data using GraphPad Prism software version 9.2.0 (GraphPad software, San Diego, CA, USA). We performed experiments at least in triplicate.

## 3 RESULTS

### 3.1 Aristolochic Acid I (AAI) exposure caused toxicity in HK2 proximal tubule cells

Aristolochic acid I (AAI) is present in *Aristolochia clematitis* (Figure 1A,D). HK2 cells exposed to AAI for 24 h at 40 μM concentration (Figure 1C) showed marked morphological changes compared to untreated cells (Figure 1B). We found a dose/ time‐dependent response to AAI exposure on the proliferation of HK2 cells (one‐way anova ****p < 0.0001, *n* = 6; Figure 1E, F). AA‐induced time‐dependent decrease in HK2 cells proliferation was further confirmed by BrdU staining (One‐way anova **p = 0.0015, *** *p* = 0.0001, *n* = 4; Figure 1G,H). AAI exposure decreased the viability of proximal tubule cells in a time‐ and concentration‐dependent manner (one‐way anova ****p < 0.0001 for 40 μM and 24 h exposure, *n* = 6; Figure 1I,J). Cell toxicity also increased with increasing AAI exposure time (one‐way anova ****p < 0.0001, *n* = 6; Figure 1L); however, cytotoxicity decreased upon increasing AAI concentration (Figure 1K), which may be due to increased necrosis at higher concentrations of AAI. We quantified apoptosis by the ratio of caspase 3/7 and found that apoptosis significantly increased at and above 40 μM concentration of AA (one‐way anova ****p < 0.0001, *n* = 6; Figure 1M) and at 24 h exposure (one‐way anova ****p < 0.0001, *n* = 6; Figure 1N). Increased AA‐induced apoptosis was further confirmed through flow cytometry using propidium iodide (PI) and Annexin V‐FITC (Figure 1O,P). We also found that AA‐exposed HK2 cells significantly underwent G2M cell cycle arrest (Figure 1Q,R). Although AA exposure increased apoptosis and G2/M cell cycle arrest in same time‐dependent manner, however, superimposing both data showed that cell cycle arrest at G2/M phase was not the causation of apoptosis initiation (Figure 1S).

### 3.2 AAI enhanced expression of TLRs and inflammatory cytokines

**FIGURE 2:**
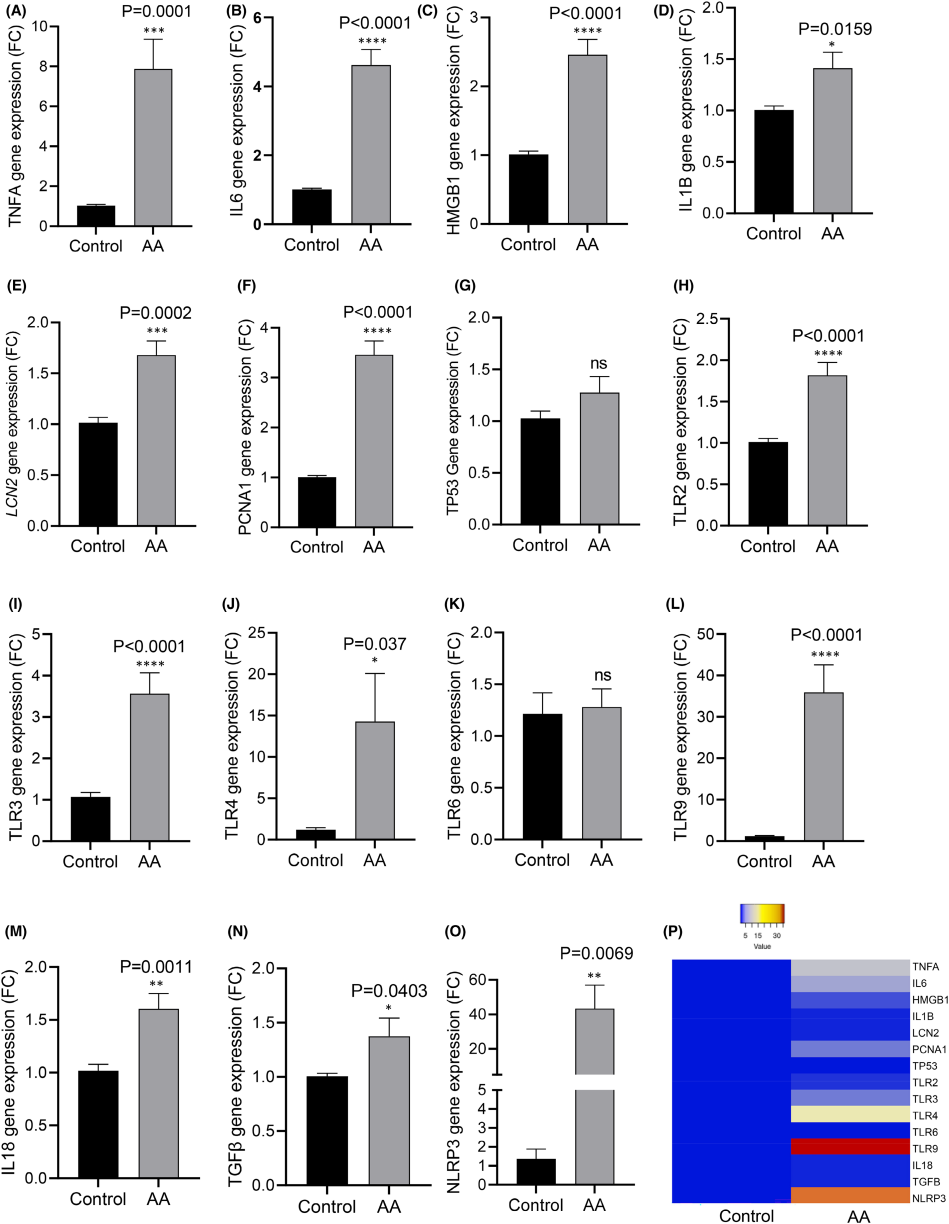
Candidate gene expressions that contribute to injury (A‐O) in HK2 cells exposed to aristolochic acidI (40 μM) compared to unexposed cells (control) showed increased expressions of TLRs (2–4, 9) and inflammatory cytokines (*TNFα, IL6, IL1B, IL18, TGFβ* and *NLRP3*)); Unpaired *t*‐test (two‐tailed); *n* = 12; *N* = 4; Heatmap of gene expressions (P) showing TLR9 as the most prominent gene overexpressed in HK2 cells upon AA exposure (40 μM) for 24 h

**FIGURE 3:**
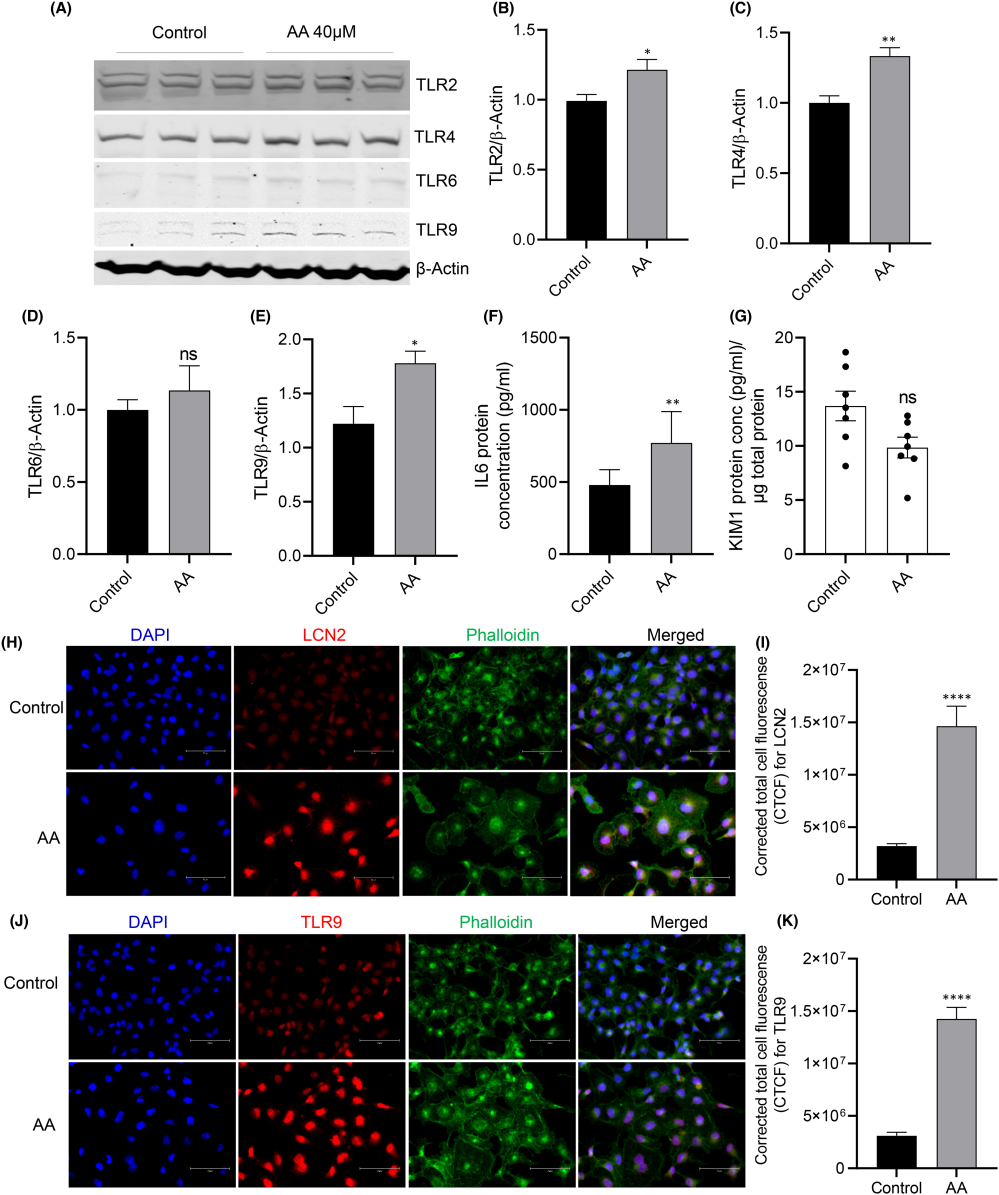
TLRs (2, 4, 6, 9) protein expressions were increased in HK2 cells after exposure to AAI (40 μM) for 24 h (A); B: TLR2, Unpaired *t*‐test *p value = 0.0329; C: TLR4, *n* = 5, Unpaired *t*‐test **p value = 0.0017; D: TLR6, NS = not significant; E: TLR9, *n* = 5; Unpaired *t*‐test *p value = 0.0165; *n* = 6; F: IL6 ELISA **p = 0.001; Two tailed *p* value, Ratio paired *t*‐test; *N* = 8; G: KIM1 ELISA, *p* = ns; AAI exposure (40 μM) increased expression of LCN2 (H) and TLR9 (J) proteins compared to unexposed HK2 cells; cell fluorescence measurement of LCN2 (I) and TLR9 (K); Unpaired *t*‐test ****p < 0.0001

We performed candidate gene expression analysis for AKI‐associated genes based on our previous publication^23^ in HK2 cells exposed to AAI in comparison with unexposed cells (control). We found significantly increased expressions of TLRs (2–4, 9, and) and inflammatory cytokines (*TNFα, IL6, IL1B, IL18, TGFβ* and *NLRP3*) in HK2 cells after exposure to AAI (40 μM) for 24 h (unpaired *t*‐test *p* < 0.05; *n* = 12; Figure 2A–O). Heat plot of gene expression revealed markedly increased expression of *TLR 9* in AA‐exposed tubule cells (gene expression fold change >30; *p* < 0.0001; Figure 1L,P). Western blot analysis confirmed significantly increased TLR 9 (unpaired *t*‐test *p = 0.0165; *n* = 6), TLR 2 (unpaired *t*‐test *p* = 0.0329; *n* = 5) and TLR 4 (unpaired *t*‐test *p* value = 0.0017; *n* = 5) in AA (40 μM) exposed HK2 cells (Figure 3A–E). ELISA showed that IL6 levels were significantly higher in the supernatant of AAI‐exposed HK2 cells compared to unexposed cells (770 pg/mL vs 479 pg/mL in control; *p* = 0.001; Figure 3F) although KIM1 protein concentration was not significantly changed in similar conditions (Figure 3G). Immunofluorescence confirmed that another KI marker LCN2, and TLR9 protein expression was increased in HK2 cells after exposure to AAI (40 μM) for 24 h (Figure 3H–K; corrected for total cell fluorescence *p* < 0.0001).

### 3.3 ROS production and mitochondrial dysfunction in HK2 cells upon AAI exposure

**FIGURE 4:**
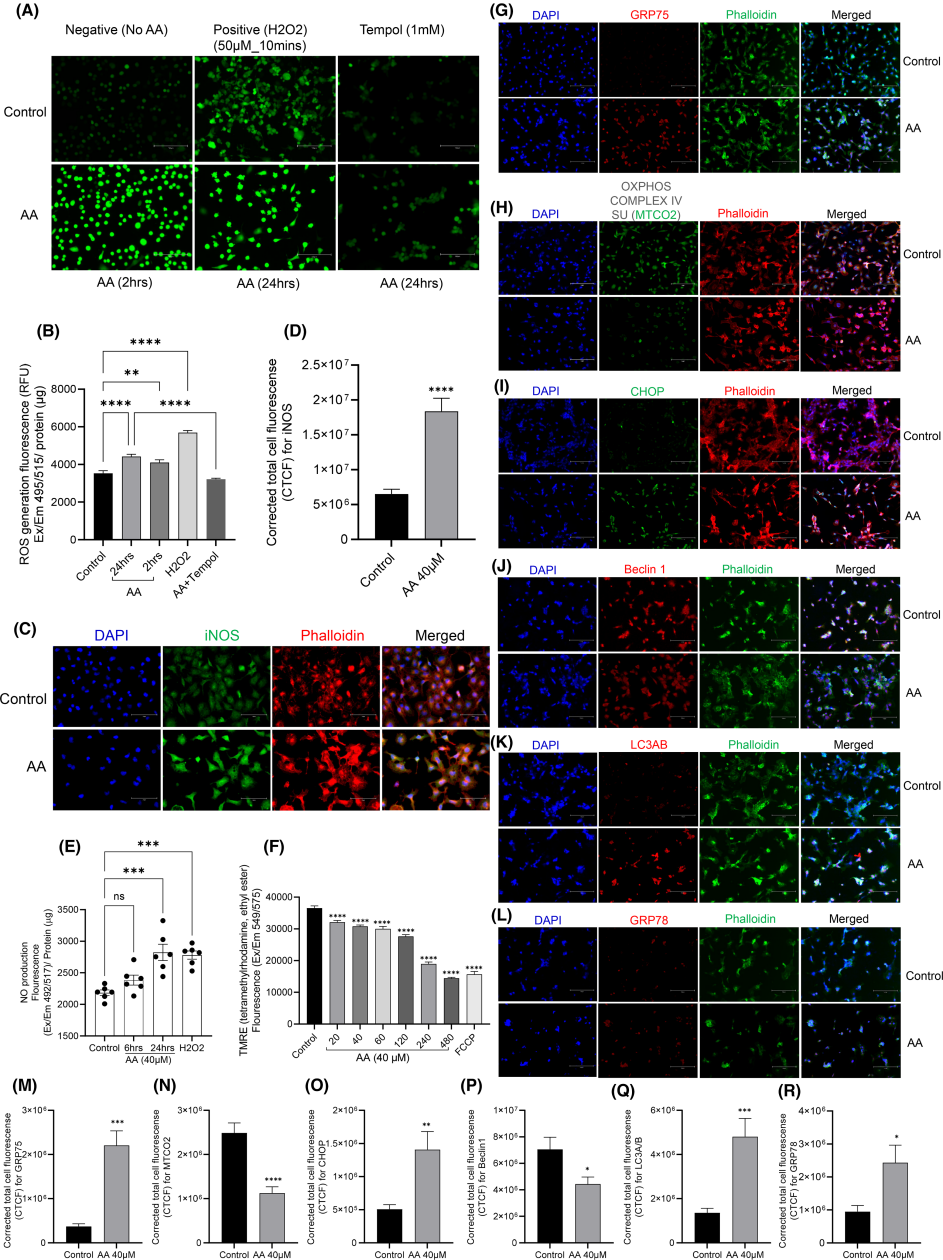
ROS production and mitochondrial dysfunction in HK2 cells upon AAI (40 μM) exposure. A: AA (40 μM) increased ROS generation in HK2 cells (20x images) as early as 2 h and persisted for up to 24 h compared with unexposed HK2 cells (control) and H2O2 (50 μM for 10 mins); B: ROS fluorescence measurement data; *p = 0.0127; One‐way anova test with Dunnett's multiple comparisons test; iNOS expression and NO production was increased in HK2 cells exposed with AAI (40 μM); C: Immunofluorescence of iNOS (green), phalloidin (red) and nucleus (blue) D: corrected cell fluorescence (CTCF) for iNOS, Unpaired *t*‐test ****p < 0.0001. E: NO production, one‐way anova Dunnett's multiple comparisons test **p = 0.0011, ****p < 0.0001; *N* = 6; F: AA (40 μM) exposure causes loss of mitochondrial membrane potential (TMRE assay); one‐way anova with Dunnett's multiple comparisons test ****p < 0.0001; *N* = 6; Comparison of immunofluorescence between control vs AA (40 μM, 24 h) exposed HK2 cells: GRP75 (G), CTCF for GRP75, unpaired *t*‐test ***p = 0.0003 (M); MTCO2 (H), CTCF for MTCO2, unpaired *t*‐test ****p < 0.0001 (N); CHOP (I), CTCF for CHOP, unpaired *t*‐test **p = 0.0051 (O), Beclin1 (J), CTCF for Beclin1, unpaired *t*‐test ***p = 0.0003 (P); LC3A/B (K), CTCF for LC3A/B, unpaired *t*‐test ***p = 0.0008 (Q) and GRP78 (L), CTCF for GRP78, unpaired *t*‐test *p = 0.017 (R)

AAI (40 μM) exposure significantly increased ROS generation in HK2 cells as early as 2 h, which was sustained for 24 h compared with unexposed HK2 cells (control). Pre‐treatment with tempol, a radical scavenger and metal‐independent superoxide dismutase‐mimetic, reversed the oxidizing effects of AAI in HK2 cells (Figure 4A,B; *p* = 0.0127).

We found that iNOS expression increased in HK2 cells exposed to AAI (CTCF for iNOS, unpaired *t*‐test *p* < 0.0001; Figure 4C,D) along with a significant increase in NO production (Figure 4E). Increased ROS and NO production may cause mitochondrial dysfunction; therefore, we measured mitochondrial membrane potential through TMRE assay and found that AAI (40 μM) exposure causes loss of mitochondrial membrane potential significantly (Figure 4F; one‐way anova Dunnett's multiple comparisons test ****p < 0.0001). Mitochondrial damage was further confirmed by checking protein expression of associated markers (GRP75, MTCO2, CHOP, LC3A/B, GRP78 and Beclin1) through immunofluorescence (Figure 4G‐L). We found upregulation of GRP75 (CTCF unpaired *t*‐test ***p = 0.0003; Figure 4M), CHOP (CTCF unpaired *t*‐test **p = 0.0051; Figure 4O), LC3A/B (CTCF unpaired *t*‐test ***p = 0.0008; Figure 4Q) and GRP78 (CTCF unpaired *t*‐test *p = 0.017; Figure 4R); however, downregulation of protective markers such as MTCO2 (CTCF unpaired *t*‐test ****p < 0.0001; Figure 4N) and Beclin1 (CTCF unpaired *t*‐test ***p = 0.0003; Figure 4P).

### 3.4 AAI induced fibrosis/ epithelial‐to‐mesenchymal transition in HK2 cells

**FIGURE 5:**
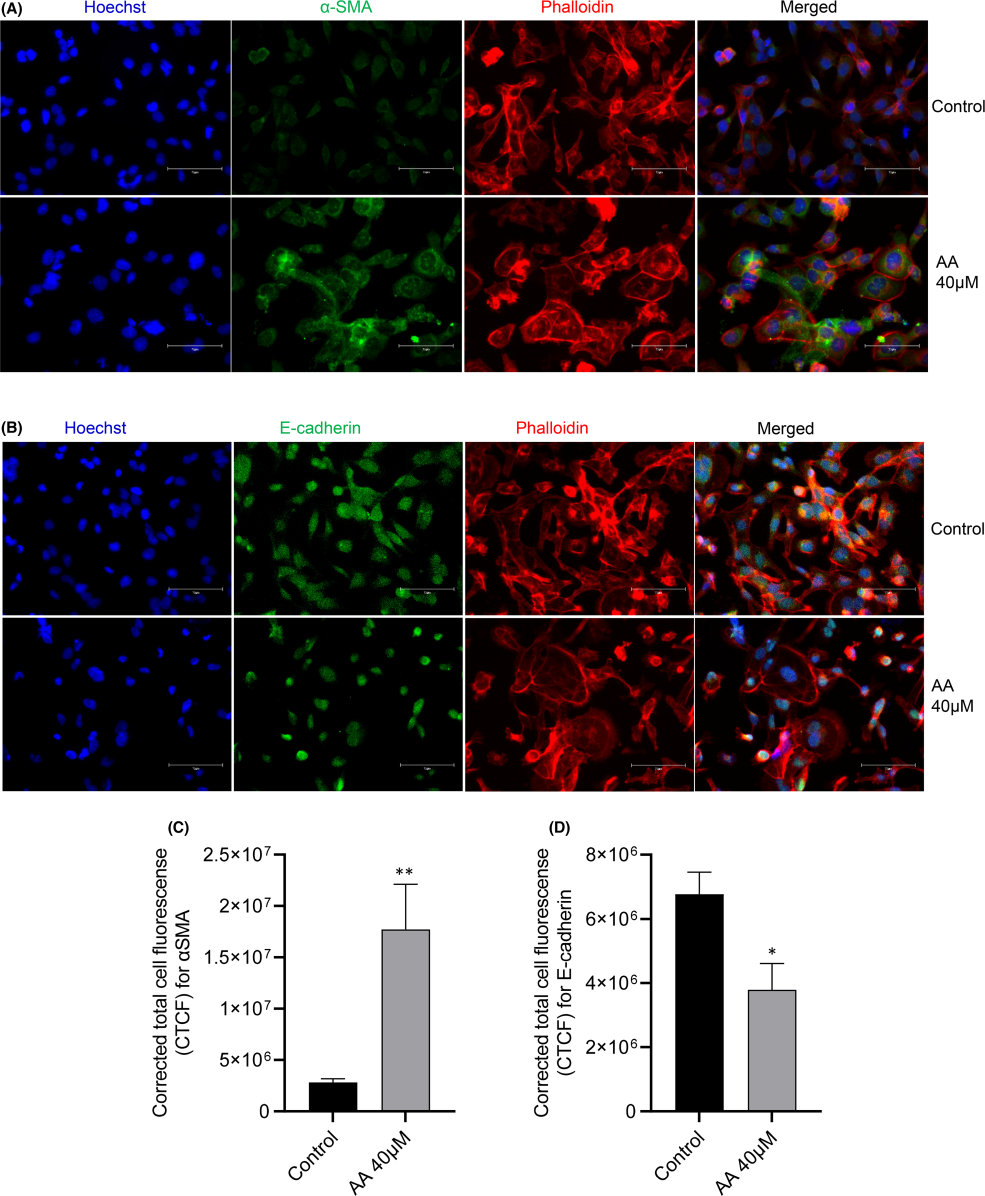
AAI induces fibrosis/ epithelial‐to‐mesenchymal transition (EMT) in HK2 cells in 24 h; A: AA (40 μM) increased α‐SMA expression (green) in HK2 cells compared to unexposed HK2 cells; B: E‐cadherin expression decreased in AA‐treated cells; C: corrected total cell fluorescence (CTCF) for α‐SMA in AA‐exposed vs unexposed HK2 cells, Unpaired *t*‐test **p = 0.0035; D: CTCF for E‐cadherin in AA‐exposed vs unexposed HK2 cells, Unpaired *t*‐test *p = 0.0121

AA nephropathy is characterized by extensive fibrosis’ we, therefore, tested whether AAI can induce EMT through indicator markers α‐SMA and E‐cadherin in AA‐exposed HK2 cells. Our data showed significant upregulation of α‐SMA (Figure 5A,C; unpaired *t*‐test **p = 0.0035) and downregulation of E‐cadherin (Figure 5B,D; unpaired *t*‐test *p = 0.0121). Data were normalized with phalloidin as a non‐specific protein.

### 3.5 Pretreatment with 1 mM tempol and TLR 9 knock‐down ameliorated AA‐induced overexpression of KI markers in HK2 cells

**FIGURE 6:**
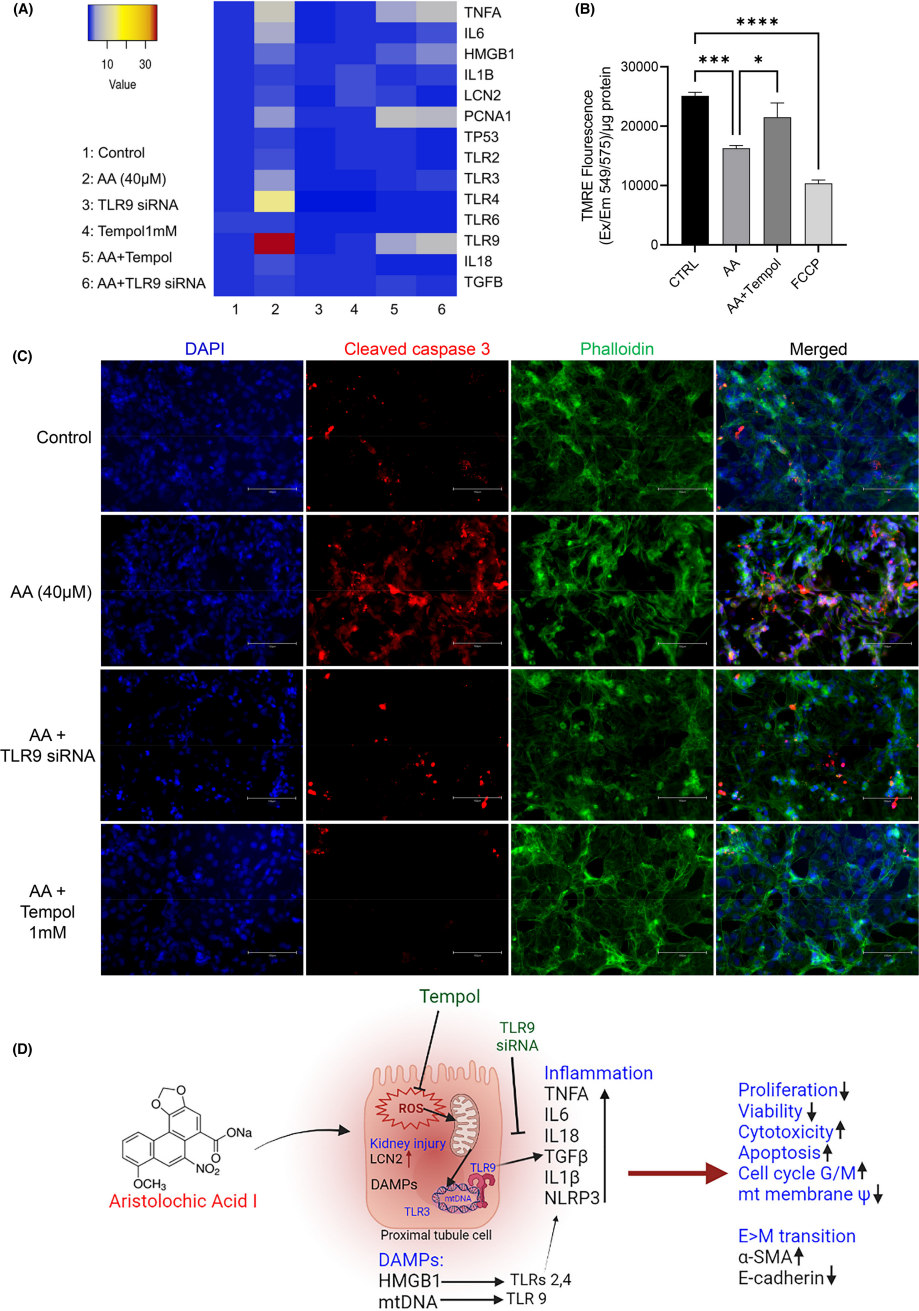
Tempol pre‐treatment and TLR9 KD ameliorated AAI‐induced injury in HK2 cells. A: Heatmap of gene expressions showing pretreated HK2 cells with tempol (1 mM) and TLR9 siRNA ameliorated AAI (40 μM, 24 h) induced overexpression of KI markers; B: Tempol (1 mM) pre‐treatment reduces AA (40 μM, 24 h) induced mitochondrial membrane potential loss; One‐way anova ***p = 0.0003, ****p < 0.0001, *p = 0.0282; C: Immunofluorescence of cleaved caspase 3 (RED) increased in AA (40 μM, 24 h) exposed HK2 cells than untreated cells which reduced upon knocking down TLR9 and tempol (1 mM) pre‐treatment before AA exposure; D: Graphical summary of mechanism of AAI‐induced injury to proximal tubules and amelioration by tempol/ TLR9‐siRNA

Our data indicated a possible mechanism of AA‐induced toxicity in HK2 cells via ROS, mt –DNA, TLR9 axis. Therefore, we tried to rescue HK2 cells by inhibiting ROS generation through tempol (1 mM) pre‐treatment and TLR 9 siRNA. We found that both tempol and TLR 9 siRNA ameliorated AA‐induced overexpression of KI markers (Figure 6A). We also found that TLR9 KD and tempol pretreatment can reduce AA‐induced mitochondrial membrane potential loss (Figure 6B) and apoptosis in HK2 cells (Figure 6C).

## 4 DISCUSSION

Aristolochic acid nephropathy (AAN) causes a rapidly progressive interstitial nephritis, which leads to end‐stage renal disease. AAN was originally reported in Belgium in a group of patients who had ingested slimming pills containing powdered root extracts of Chinese herbs^3^, ^10^, ^28^ and initially called Chinese‐herb nephropathy (CHN). Later, investigations revealed that it was due to the conflation of similar names (Han Fang Ji and Guang Fang Ji) of two herbs (*Stephania tetrandra* and *Aristolochia fangchi*) in Chinese and due to erroneous botanical classification.^29^ AA is implicated as a cause of Balkan‐endemic nephropathy (BEN), which is endemic in Serbia, Bosnia, Croatia, Bulgaria and Romania, where *Aristolochia clematitis* is widely present,^30^, ^31^ although there is some controversy.^32^, ^33^, ^34^ What is noncontroversial, however, is that AA can cause a rapidly progressive kidney disease often associated with a high incidence of upper urothelial cancer and is responsible for many cases through either use as herbal medicine or food contaminant throughout the world. The true incidence of AAN is unknown. New cases of AAN continue to be reported worldwide despite the Food and Drug Administration's warnings regarding the safety of botanical remedies containing AA, and plants containing AA are still available via the Internet.^31^, ^35^

Previous studies investigating the mechanisms of AAN suggested that AAI acts mainly on proximal renal tubular epithelial cells^36^, ^37^ activating MAPK/extracellular signal‐regulated kinase 1/2 (MEK/ERK1/2) signalling pathway, followed by depletion of intracellular GSH,^38^ NQO1, CYP1A1/2, reduced organic anion transporters (OAT), inhibition of PI3K‐Akt signalling, and activation of NF‐κB, mitophagy, Rap1 signalling and TCA cycle.^31^, ^37^, ^38^, ^39^, ^40^, ^41^, ^42^ Several reports have proposed a role for the organic anion (OA) transporter (OAT) family in AA‐mediated nephrotoxicity.^43^, ^44^ Recent evidence indicates that AA is concentrated within proximal tubular epithelial cells via the basolateral OAT (OAT1), which exchanges organic anions such as para‐aminohippurate (PAH) and AA for α‐ketoglutarate.^45^, ^46^

The integrative omics analysis clearly demonstrated that AAI damage to HK‐2 cells involves three pathways: the amino sugar and nucleotide sugar metabolism pathway, the amino acid biosynthetic pathway and the carbon metabolism pathway. Through catabolism of amino acids, cells can use carbon skeletons to form tricarboxylic acid cycle (TCA) cycle intermediates used in the central metabolic pathway.^42^ Carbon metabolism is one of the basic metabolic pathways of organisms and can affect many physiological functions.^47^, ^48^

TLRs are well‐known for their role as first responders to exogenous/ endogenous damage signals; however, the involvement of TLRs in AAN has not been systematically investigated. In the present study, we demonstrated that AAI exposure increased ROS generation in HK2 cells, which induced mitochondrial dysfunction and activation of endogenous alarmins (DAMPs) such as HMGB1 and mt DNA. TLRs 2, 4 and 9 were activated by their ligands HMGB1 and mt DNA, respectively, and activated downstream inflammatory pathways. ROS inhibition by tempol and TLR9 knock‐down through siRNA transiently ameliorated the toxic effect of AAI.

We detected significantly increased mRNA expressions of TLRs 2,3,4 and 9 along with HMGB1 in HK2 cells exposed to AAI. HMGB1 is a known ligand of TLRs 2,4, and previously, we have shown that HMGB1 regulates activation of TLRs 2, 4 and 6 in proximal tubule cells.^23^ Therefore, AAI‐induced nephropathy may be initiated through HMGB1‐mediated TLR activation. Furthermore, in the present study, we identified markedly higher expression of TLR 9 (>30‐fold) in AAI‐exposed HK2 cells compared to unexposed cells. TLR 9 is a cytosolic receptor that recognizes unmethylated CpG DNA found in microbial DNA, DNA viruses and endogenous mt DNA.^49^, ^50^, ^51^ TLR 9 activation is complex and can have different roles in different cell types. For example, in renal tubular epithelial cells, TLR 9 promotes cell injury and death whereas TLR 9 signalling in other cell types may promote cytoprotective effects.^52^ Since the cells in this study were endotoxin free and experiments were performed in aseptic conditions, we suspected mitochondrial DNA as a ligand for TLR 9 activation and detected mitochondrial membrane potential loss in HK2 cells upon AA1 exposure through TMRE assay. Thus, not only HMGB1 but mt DNA also acts as a DAMP ligand (and probably to a higher extent) to activate TLRs in AAN.

Renoprotective effect of Stat1 deletion was shown in a murine model of aristolochic acid nephropathy.^53^ Similarly, our previous study^23^ showed STAT1 knockdown can protect PTCs through decreasing HMGB1 release. In the present study, we found that AA‐induced significant overexpression of HMGB1 mRNA (Figure 2B) compared to control HK2 cells along with TLRs 2,3,4,9. This suggests that AA‐induced overexpression of HMGB1 and TLRs 2,3,4,9 is mediated via STAT1 activation. A recent report showing the macrophage interferon regulatory factor 4 deletion ameliorates aristolochic acid nephropathy is consistent with our observation on the role of innate immunity in AA nephropathy.^54^ The study confirms the expectation that anti‐TLR strategy may be of therapeutic value.

AAI induced and DAMPs (HMGB1/mt DNA) mediated activation of TLR‐initiated downstream inflammatory cascade (IL6, TNFA, IL1B, IL18, TGFB and NLRP3), shown at mRNA and protein levels (Figures 2, 3). Furthermore, there were morphological changes including reduced proliferation, cell viability, increased cytotoxicity, G2/M cell cycle arrest and apoptosis in HK2 cells (Figure 1). Increased ROS generation, iNOS expression and NO production were present in AAI‐exposed HK2 cells (Figure 4), leading to proximal tubule injury confirmed by the markedly increased expression of kidney injury marker LCN2 in AAI‐exposed cells (Figure 2E, 3H). Proximal tubules contain abundant mitochondria to perform the energy‐demanding processes of reabsorption and secretion. Mitochondria are the most active organelles in the cell where approximately 90% of the total oxygen content in the cell is consumed to enable oxidative phosphorylation and ATP synthesis^55^ and therefore more susceptible to ROS‐mediated injury. Damaged or dysregulated mitochondria generate excessive ROS and start a chain reaction damaging other mitochondria.^56^ NO is a reactive nitrogen species (RNS) that is generated from injured mitochondria by inducible isoform of nitric oxide synthase (iNOS or NOS2) enzyme, and it can alter respiration, mitochondrial biogenesis and oxidative stress through increased production of ROS/RNS and thus can impact cell physiology.^56^, ^57^ Our data suggest that AAI induces DAMPS that bind to specific TLRs and initiate a pro‐inflammatory cascade by damaging mitochondria and generating ROS as well as RNS.

Additionally, we found that AA exposure leads to decreased expression of E‐cadherin and increased expression of α‐SMA in PTCs, indicating phenotypic transformation of epithelial cells to a myofibroblast phenotype through epithelial‐to‐mesenchymal transition (EMT). Although fibrosis has generally been associated with increased expression of TGFβ‐dependent ECM components,^58^ it can also be linked to overexpression of TLR 9. TLR 9 depletion can attenuate renal tubulointerstitial fibrosis after I/R injury.^59^ Similarly, TLR9 overexpression is associated with pulmonary fibrosis and is an indicator of fibrosis.^60^ Our findings showed that HK2 cells could be rescued through ROS inhibitor (tempol 1 mM) and TLR 9 siRNA by disrupting the ROS‐DAMP‐TLR9 axis. These observations suggest potential antifibrotic strategies in the kidney and other organs. In the future, other known inhibitors of TLR 9 could be used to inhibit downstream cytokine cascade or FDA‐approved drugs could be screened for their potential role in AAN.

In conclusion, this study confirms that AAI is a potent nephrotoxin primarily affecting PTCs. Our study suggests the role of oxygen radicals, DAMPs (HMGB1/mt DNA) mediated TLR activation and inflammatory response in AA‐induced cytotoxicity, which could be ameliorated by ROS inhibitor (tempol 1 mM) and TLR 9 siRNA. Extensive fibrosis is a hallmark of AA nephrotoxicity and our study showing a prominent TLR 9 effect in AA‐exposed PTCs indicate a major role for innate immunity in AA‐induced fibrogenesis in the kidney.

## AUTHOR CONTRIBUTIONS

RU and VB conceived and designed research; RU performed experiments, analysed data and prepared figures; RU and VB interpreted results of experiments, drafted manuscript, edited/revised manuscript and approved the final version of manuscript.

## CONFLICT OF INTEREST

The authors confirm that there are no conflicts of interest.

## Data Availability

The data that support the findings of this study are available from the corresponding author upon reasonable request.
